# *Azospirillum brasilense* promotes increases in growth and nitrogen use efficiency of maize genotypes

**DOI:** 10.1371/journal.pone.0215332

**Published:** 2019-04-18

**Authors:** Douglas Mariani Zeffa, Luiz Júnior Perini, Mayara Barbosa Silva, Nicholas Vieira de Sousa, Carlos Alberto Scapim, André Luiz Martinez de Oliveira, Antônio Teixeira do Amaral Júnior, Leandro Simões Azeredo Gonçalves

**Affiliations:** 1 Department of Agronomy, Universidade Estadual de Maringá, Maringá, Paraná, Brazil; 2 Department of Agronomy, Universidade Estadual de Londrina, Londrina, Paraná, Brazil; 3 Department of Biochemistry and Biotechnology, Universidade Estadual de Londrina, Londrina, Paraná, Brazil; 4 Plant Breeding Laboratory, Universidade Estadual do Norte Fluminense Darcy Ribeiro, Campos dos Goytacazes, Rio de Janeiro, Brazil; University of Delhi, INDIA

## Abstract

The development of cultivars with an improved nitrogen use efficiency (NUE) together with the application of plant growth-promoting bacteria is considered one of the main strategies for reduction of fertilizers use. In this sense, this study: i) evaluated the effect of *Azospirillum brasilense* on the initial development of maize genotypes; ii) investigated the influence of *A*. *brasilense* inoculation on NUE under nitrogen deficit; and iii) sought for more NUE genotypes with higher responsiveness to *A*. *brasilense* inoculation. Twenty-seven maize genotypes were evaluated in three independent experiments. The first evaluated the initial development of maize genotypes with and without *A*. *brasilense* (strain Ab-V5) inoculation of seeds on germination paper in a growth chamber. The second and third experiments were carried out in a greenhouse using Leonard pots and pots with substrate, respectively, and the genotypes were evaluated at high nitrogen, low nitrogen and low nitrogen plus *A*. *brasilense* Ab-V5 inoculation. The inoculation of seeds with *A*. *brasilense* Ab-V5 intensified plant growth, improved biochemical traits and raised NUE under nitrogen deficit. The inoculation of seeds with *A*. *brasilense* can be considered an economically viable and environmentally sustainable strategy for maize cultivation.

## Introduction

The world yield and productivity of maize (*Zea mays* L.) doubled in the last three decades, resulting in an output of 1,034.8 million tons of grain in 2017/2018 [1]. This significant yield increase is attributed mainly to chemical fertilizers, breeding and crop management [2]. However, the dependence of modern agriculture on chemical fertilizers is alarming, since the indiscriminate use of these inputs has been causing serious environmental problems, e.g., water eutrophication, soil acidification and air pollution [3–5].

With regard to nitrogen (N) fertilizers, more than 100 million tons N year^-1^ are produced industrially based on fossil energy sources [6,7]. Apart from the environmental problems, N fertilizers also account for 15 to 20% of the production costs of maize [8]. It is estimated that, without N fertilizers, the global food production would be sufficient for less than half the current population of 7.6 billion people [9].

Among the abiotic factors, N deficit is seen as one of the main limiting factors, since N is not only the most demanded nutrient but also the element with greatest influence on maize productivity [10]. Although maize depends heavily on N fertilization, the nitrogen use efficiency (NUE), in other words, grain yield or biomass produced per unit of soil available N, is estimated at < 50% [11,12]. According to Ladha et al. [2], the two most promising strategies to reduce dependence on N fertilization in maize cultivation are: i) develop plants with increased NUE and ii) application of associative diazotrophic bacteria to improve non-symbiotic N fixation. These associative diazotrophic bacteria may play an important role in plant nutrient uptake, acting as biofertilizers, phytostimulators and mitigators of biotic and abiotic stresses [13,14]. Among the diazotrophic bacteria associated with different species of agricultural importance, the most important genera are *Arthrobacter*, *Azobacter*, *Azospirillum*, *Bacillus*, *Burkholderia*, *Clostridium*, *Gluconacetobacter*, *Herbaspirillum* and *Pseudomonas* [15–17].

The genus *Azospirillum* [18] includes a group of bacteria that can be either associated with the plant rhizosphere, in external colonization, or associated endophytically if the intercellular spaces of the roots are colonized [19]. According to the List of Prokaryotic Names with Standing in Nomenclature [20], 19 *Azospirillum* species have been described, considered the best-studied genus of plant growth-promoting bacteria (PGPB) [21]. Among the main species of the genus are *A*. *brasilense*, *A*. *lipoferum*, *A*. *halopraeferens* and *A*. *oryzae*, which are widely used as biofertilizers, in particular of cereals. According to Pereg et al. [22], *Azospirillum* is associated with more than 113 plant species of 35 botanical families and can be considered a genus with a broad spectrum of plant affinity.

Bacteria of the genus *Azospirillum* are capable promote the plant growth by different mechanisms, including the biosynthesis and release of amino acids, indo-acetic acid, cytokinins, gibberellins and other polyamines, favoring root growth and, consequently, intensifying water and nutrient uptake by plants [23–25]. Aside from these benefits, *Azospirillum* has the ability to fix atmospheric nitrogen (N_2_) through the biological nitrogen fixation process (BNF) and can therefore directly contribute to make N available to non-leguminous species [26,27]. In this context, it may not be only one mechanism that is responsible for the full growth-promoting effect to *Azospirillum*, and the effect of these bacteria could be better explained by the "Multiple Mechanism Theory" formulated by Bashan and Levanony [28], which assumes that several factors may be implicated in the successful *Azospirillum*–plant association [23].

The identification of maize genotypes with superior NUE associated with *Azospirillum* inoculation can be considered important strategy to overcome low yields of maize cultivate under N deficit. Thus, the objectives of this study were to i) evaluate the effect of *A*. *brasilense* on the initial development of maize genotypes to identify those most responsive this PGPB; ii) determine the influence of *A*. *brasilense* inoculation on NUE of maize grown under N deficit; and iii) identify maize inbred lines with higher NUE that are more responsive to *A*. *brasilense* inoculation.

## Materials and methods

### Biological material

Twenty-seven maize genotypes were evaluated, including 26 inbred lines of the germplasm bank from Universidade Estadual de Maringá (UEM) and the single-cross hybrid 2B587PW (Dow AgroSciences). The inbred lines were obtained by successive self-pollinations from different maize commercial hybrids. The bacterial strain *A*. *brasilense* Ab-V5 was used in the experiments. This strain is derived from a selection program that evaluated N_2_-fixing capacity *in vitro* and under field conditions in Paraná State, Brazil, being highly efficiency in promoting growth of maize in several trials, mainly due to capacity of producing phytohormones, increasing root growth and nutrients uptake [29]. The *A*. *brasilense* Ab-V5 is registered for commercial use in Brazil by the Ministry of Agriculture, Livestock and Food Supply (MAPA), and is part of the "Collection of Diazotrophic Bacteria and Plant Growth Promoters" of Embrapa Soybean, Londrina, Paraná, Brazil.

### Inoculant preparation

The inoculant was prepared from a pre-inoculum in DYGS liquid medium [30] and incubated on a rotary shaker (180 rpm) at 28±2°C for 24 h. The pre-inoculum was multiplied in Erlenmeyer flasks with 250 mL of Form 15 culture medium [31] and incubated in an orbital shaker (180 rpm) at 28±2°C for 24 h. After the growth period, the bacterial population density was diluted to a concentration standard of 1 × 10^8^ mL^-1^ cells.

### Experiment on germination paper

For the experiment on germination paper (E1), a completely randomized design with four replications was used, evaluating 27 maize genotypes with (+Azo) and without (−Azo) inoculation with *A*. *brasilense* Ab-V5. The seeds were initially disinfected by immersion in 95% (v/v) ethanol solution for 30 sec, followed by soaking in 5% (v/v) H_2_O_2_ solution for 10 min, and then washed six times with sterile deionized water [32]. Thereafter, maize seeds from the +Azo treatments were inoculated by briefly soaking the seeds on inoculant solution to a final concentration of 3.3 × 10^−6^ cells of *A*. *brasilense* per seed. After inoculation, 30 seeds per treatment were placed on germination paper moistened with sterilized distilled water and incubated in a growth chamber at 25±2°C and 70% relative humidity.

Ten days after sowing (growth stage V1), the roots of five seedlings were scanned at 300 dpi and the images treated and analyzed with software GiA Roots [33]. The total root surface area (RSA, in cm^3^) and total root length (RL, in cm) were evaluated. The shoot part and root system of the seedlings were oven-dried separately under forced ventilation at 60°C for 72 h to determine shoot dry mass (SDM, in g) and root dry mass (RDM, in g).

Five other seedlings were harvested and ground for 5 min in a mortar with extraction buffer containing 5 mL sodium borate (50 mM), 2-mercaptoethanol (5 mM) and 5% polyvinylpolypyrrolidone (PVPP) (w/v), at pH 8.5 [34]. The crude extract was centrifuged at 9,000 g × 30 min at 4°C, and 4 mL supernatant was collected to assess indole-3-acetic acid (IAA, in μg g^-1^ root), total soluble proteins (PRO, in mg g^-1^ root protein), phenylalanine ammonia-lyase activity (PAL, in μmol methylcatecholine min^-1^ mg^-1^ root) and polyphenoloxidase activity (PPO, in μmol phenylalanine min^-1^ mg^-1^ root).

The traits IAA and PRO were evaluated by methodologies described by Bautista and Gallardo [35] and Bradford [36], respectively. For IAA, a 600 μL aliquot of the supernatant was mixed with 200 μL sodium borate buffer solution (50 mM) and 1.2 mL Salkowski solution and maintained for 30 min in the dark. To determine PRO, an aliquot of supernatant (50 μL) was added with 950 μL sodium borate buffer solution (50 mM) and 1 mL Coomassie Brilliant Blue G-250 reagent, after gentle shaking and a rest period of 5 min. Readings on an Agilient 8453 spectrophotometer (Agilient Technologies, USA) were performed at wavelengths of 540 and 595 nm, respectively, for IAA and PRO.

The activities of the enzymes PAL and PPO were assessed by the methodologies described by Kamdee et al. [37] and Sommano [38], respectively. To determine PAL, an aliquot of the supernatant (150 μL) was mixed in 3 mL sodium borate buffer solution (50 mM) and 350 μL L-phenylalanine (100 mM). Subsequently, the test tubes were vortexed and incubated at 40°C for 1 h. The PPO activity was determined by adding 100 μl supernatant to 250 μl 4-methylcatechol (10 mM) and 650 μl potassium phosphate buffer (0.1 M). Thereafter, the test tubes were vortexed and incubated at 30°C for 30 min. Spectrophotometric readings were performed at wavelengths of 290 and 410 nm, respectively, for PAL and PPO.

### Experiment in Leonard pots

The experiment was arranged in a completely randomized design with four replications and the 27 maize genotypes were evaluated in three conditions: cultivation at high nitrogen (HN), low nitrogen (LN) and LN plus *A*. *brasilense* Ab-V5 inoculation (LN+Azo). After disinfestation, the seeds on moist germination paper were incubated in a growth chamber at 25±2°C and 70% relative humidity. After five days, the seedlings were selected for uniformity of length, and one seedling per pot was transplanted into independent Leonard pots [39]. In condition LN+Azo, *A*. *brasilense* Ab-V5 inoculation with 1 mL inoculant per pot containing 1 × 10^8^ mL^-1^ cells was performed immediately after transplanting.

The Hoagland and Arnon [40] nutrient solution, modified by Chun [41], was used during the experimental period. In condition HN, the nutrient solution contained 2.0 mmol L^-1^ Ca (NO3)_2_; 0.75 mmol L^-1^ K_2_SO_4_; 0.65 mmol L^-1^ MgSO_4_; 0.1 mmol L^-1^ KCl; 0.25 mmol L ^-1^ KH_2_PO_4_; 1 × 10^−3^ mmol L^-1^ H_3_BO_3_; 1 × 10^−3^ mmol L^-1^ MnSO_4_; 1 × 10^−4^ mmol L^-1^ CuSO_4_; 1 × 10^−3^ mmol L^-1^ ZnSO_4_; 5 × 10^−6^ mmol L^-1^ (NH_4_)_6_Mo_7_O_24_ and 0.1 mmol L^-1^ Fe-EDTA at pH 6.0. Under LN, the nutrient solution contained 0.2 mmol L^-1^ Ca (NO_3_)_2_, i.e., the N concentration was 10 times lower than at HN, and Ca^2+^ was compensated by the addition of CaCl_2_. The other nutrients were added at the same concentration as in condition HN.

The upper part of the Leonard pots was filled with 450 cm^3^ perlite as inert substrate and the lower part (saucer) with 100 mL nutrient solution. The pots were arranged on tables under greenhouse conditions and the nutrient solution was replaced every five days. After 28 days (growth stage V4), the total root volume (RV, in cm^3^) was determined as the difference between the water volume within a graduated cylinder before and after insertion of the fresh roots. Afterwards, the shoot part and root system of the plants were stored separately in paper bags and dried in a forced ventilation oven at 60 ^o^C for 72 h for subsequent determination of the shoot dry mass (SDM, in g) and root dry mass (RDM, in g). The SDM samples were ground and used to determine total shoot nitrogen by Kjeldahl digestion method [42] using a Tecnal TE-0371 digester. The nitrogen use efficiency (NUE, in mg mg^-1^) was determined as described by Moll et al. [43] by the following formula:

$${NUE}_{ijk}=\left(\frac{{TSN}_{ijk}}{{TAN}_{ijk}}\right)\times \left(\frac{{SDM}_{ijk}}{{TSN}_{ijk}}\right)$$

where: *NUE*_*ijk*_ is the nitrogen use efficiency of genotype *i* in replication *j* under condition *k*; *TSN*_*ijk*_ is the total nitrogen contained in the shoot of genotype *i* in replication *j* under condition *k*; *TAN*_*ijk*_ is the total amount of nitrogen available for genotype *i* in replication *j* under condition *k*; and *SDM*_*ijk*_ represents the shoot dry mass of genotype *i* in replication *j* under condition *k*.

### Experiment in pots with substrate

In the experiment in pots with substrate (E3) we used the same treatments and experimental design as in experiment E2. However, after selecting healthy seedlings grown on germination paper, a 3:1 (v/v) mixture of sand and soil (Eutrophic Red Latosol) was filled in 1 L plastic containers, and one seedling per pot was planted. The soil was collected at School Farm of the Universidade Estadual de Londrina (UEL), Londrina, Paraná, Brazil. The chemical properties of the substrate were analyzed: pH (H_2_O) = 5.2; H + Al = 8.12 cmolc dm^-3^; K = 0.58 cmol dm^-3^; Ca = 3.71 cmol dm^-3^; Mg = 1.60 cmolc dm^-3^; Al = 0.37 cmolc dm^-3^; P = 2.31 mg dm^-3^; and organic matter = 1.09%. Inoculation with *A*. *brasilense* Ab-V5 in condition LN+Azo was performed immediately after transplanting, applying 1 mL inoculant per pot at 1 × 10^8^ mL^-1^ cells. The pots were placed on tables in a greenhouse and fertigation was applied every five days consisting of 100 mL per pot of the nutrient solution of Hoagland and Arnon [40], modified by Chun [41]. After 28 days (growth stage V6), the traits RV (in cm^3^), SDM (in g), RDM (in g) and NUE (in mg mg^-1^) were evaluated.

### Data analysis

The data were analyzed based on restricted maximum likelihood (REML) and best linear unbiased prediction (BLUP) with software Selegen–REML/BLUP [44]. The predicted genotypic means were calculated after testing for data normality and homogeneity by the tests of Shapiro and Wilk [45] and Hartley [46], respectively. Deviance analyses (ANADEV) were performed based on the following statistical model:

y=Xu+Zg+e

where *y* is the data vector; *u* the scale for the general mean (fixed effect); *g* the vector of the genotypic effects (assumed as random); *e* the vector of errors or residues (random); and *X* and *Z* represent the incidence matrices for *u* and *g*, respectively.

The predicted genotypic means were used in Pearson’s correlation coefficient, principal component analysis (PCA) and a heatmap based on standardized data. For the heatmap analysis, Ward's clustering method [47] based on the Euclidean distance was used. The inoculation efficiency index (IEI, in %) was calculated by the following formula:

$${IEI}_{i}=\left(1-\frac{{GMLN}_{i}}{{GMI}_{i}}\right)\times 100$$

where: *IEI*_*i*_ is the inoculation efficiency index of genotype *i*; *GMLN*_*i*_ is the predicted genotype mean of genotype *i* in the low nitrogen (LN) condition; and *GMI*_*i*_ is the predicted genotype mean of genotype *i* under LN plus inoculation with *A*. *brasilense* Ab-V5 (LN+Azo). For the statistical analyses, software R (http://www.r-project.org) was used with the packages FactoMineR [48], heatmaply [49] and ggplot2 [50].

## Results

### Experiment on germination paper

The predicted genotypic means of the inoculated (+Azo) and uninoculated (−Azo) treatments and their respective inoculation efficiency indices (IEI) are listed in Tables 1 and 2. Apart from PAL (-5.87%), the mean IEI values were positive for all evaluated traits, ranging from 1.82 (RSA) to 23.56% (PPO). For SDM, the IEI was positive for 21 genotypes, from 0.99 (L1) to 24.45% (L21), and negative for six genotypes, from -7.56 (L11) to -0.44% (2B587PW). In relation to RDM, positive IEI values were observed, ranging from 0.10 (L16) to 21.30% (L12). However, for five genotypes, the values were negative, ranging from -21.68 (2B587PW) to -0.88% (L17). In general, the IEI of the inbred lines L12, L15 and L20 were the most positively affected by *Azospirillum* inoculation, with increased values for IAA, PPO, PRO, SDM, RDM, RSA and RL (Tables 1 and 2).

**Table 1:** Predicted genotype means of indole-3-acetic acid (IAA), total soluble proteins (PRO), polyphenoloxidase (PPO), phenylalanine ammonia-lyase (PAL) and their respective inoculation efficiency indices (IEI) evaluated in 27 maize genotypes grown on germination paper without (−Azo) and with inoculation (+Azo) of *Azospirillum brasilense* Ab-V5.

Genotype	IAA (mg g^-1^)			PRO (mg g^-1^)			PPO (μmol min^-1^ mg^-1^)			PAL (μmol min^-1^ mg^-1^)		
	−Azo	+Azo	IEI (%)^1^^/^	−Azo	+Azo	IEI (%)	−Azo	+Azo	IEI (%)	−Azo	+Azo	IEI (%)
L1	28.96	29.82	2.88	0.2064	0.2714	23.95	0.0836	0.1099	23.93	0.2118	0.3103	31.74
L2	31.19	30.34	-2.80	0.2467	0.3626	31.96	0.0999	0.1468	31.95	0.2197	0.3280	33.02
L3	26.88	28.91	7.02	0.2207	0.3208	31.20	0.0894	0.1299	31.18	0.2040	0.2111	3.36
L4	26.75	28.98	7.69	0.2148	0.2801	23.31	0.0870	0.1134	23.28	0.2081	0.2208	5.75
L5	27.69	28.84	3.99	0.2112	0.2859	26.13	0.0855	0.1157	26.10	0.2240	0.2653	15.57
L6	29.57	29.19	-1.30	0.2030	0.2750	26.18	0.0822	0.1113	26.15	0.2156	0.2413	10.65
L7	28.20	29.09	3.06	0.2257	0.2982	24.31	0.0914	0.1207	24.28	0.2281	0.2325	1.89
L8	29.27	31.49	7.05	0.2188	0.2767	20.93	0.0886	0.1120	20.89	0.2409	0.3183	24.32
L9	30.38	29.91	-1.57	0.2488	0.3008	17.29	0.1007	0.1218	17.32	0.2365	0.2774	14.74
L10	27.29	30.20	9.64	0.2130	0.2819	24.44	0.0862	0.1141	24.45	0.2702	0.2913	7.24
L11	27.01	29.57	8.66	0.2351	0.3371	30.26	0.0952	0.1365	30.26	0.2496	0.2049	-21.82
L12	28.08	29.30	4.16	0.2308	0.2783	17.07	0.0935	0.1127	17.04	0.2592	0.2080	-24.62
L13	27.16	29.36	7.49	0.2007	0.3068	34.58	0.0813	0.1242	34.54	0.3303	0.2244	-47.19
L14	27.44	29.15	5.87	0.2327	0.3126	25.56	0.0942	0.1266	25.59	0.2542	0.2284	-11.30
L15	29.79	30.83	3.37	0.2289	0.3163	27.63	0.0927	0.1281	27.63	0.2322	0.2710	14.32
L16	28.30	29.50	4.07	0.2273	0.2932	22.48	0.0920	0.1187	22.49	0.2455	0.2457	0.08
L17	27.82	29.74	6.46	0.2431	0.3037	19.95	0.0984	0.1230	20.00	0.2842	0.2144	-32.56
L18	28.54	29.43	3.02	0.2080	0.2905	28.40	0.0842	0.1176	28.40	0.3001	0.2549	-17.73
L19	28.66	30.00	4.47	0.2049	0.2732	25.00	0.0830	0.1106	24.95	0.2942	0.2175	-35.26
L20	30.03	31.03	3.22	0.2451	0.3271	25.07	0.0992	0.1324	25.08	0.3477	0.2839	-22.47
L21	28.80	30.69	6.16	0.2373	0.3096	23.35	0.0961	0.1254	23.37	0.3663	0.3368	-8.76
L22	29.10	30.09	3.29	0.2240	0.2838	21.07	0.0907	0.1149	21.06	0.2889	0.2603	-10.99
L23	27.58	29.03	4.99	0.2398	0.2296	-4.44	0.0971	0.0991	2.02	0.2643	0.2370	-11.52
L24	30.75	29.65	-3.71	0.2095	0.2957	29.15	0.0848	0.1197	29.16	0.3075	0.2992	-2.77
L25	27.95	29.25	4.44	0.2225	0.2881	22.77	0.0901	0.1167	22.79	0.3170	0.2502	-26.70
L26	29.41	31.24	5.86	0.2542	0.3561	28.62	0.1029	0.1442	28.64	0.3648	0.3310	-10.21
2B587PW	28.42	30.49	6.79	0.2169	0.2271	4.49	0.0878	0.0911	3.62	0.2767	0.2016	-37.25
Mean	28.56	29.82	4.23	0.2248	0.2956	23.36	0.0910	0.1199	23.56	0.26821	0.25798	-5.87

^1/^IEI = $\left(1-\frac{-Azo}{+Azo}\right)\;x\;100$

**Table 2:** Predicted genotypic means of shoot dry mass (SDM), root dry mass (RDM), total root surface area (RSA), total root length (RL) and their respective inoculation efficiency indices (IEI) evaluated in 27 maize genotypes grown on germination paper without (−Azo) and with inoculation (+Azo) of *Azospirillum brasilense* Ab-V5.

Genotypes	SDM (g plant^-1^)			RDM (g plant^-1^)			RSA (cm^3^ plant^-1^)			RL (cm plant^-1^)		
	−Azo	+Azo	IEI (%)^1^^/^	−Azo	+Azo	IEI (%)	−Azo	+Azo	IEI (%)	−Azo	+Azo	IEI (%)
L1	0.2397	0.2421	0.99	0.1905	0.2192	13.09	202.76	220.18	7.91	28.76	37.21	22.71
L2	0.1732	0.1933	10.40	0.1785	0.1760	-1.42	182.23	207.38	12.13	26.69	31.14	14.29
L3	0.1986	0.2155	7.84	0.1932	0.2080	7.12	196.53	217.20	9.51	27.57	35.00	21.22
L4	0.1916	0.2135	10.26	0.1718	0.2015	14.74	334.24	233.28	-43.28	27.13	34.76	21.95
L5	0.2027	0.2115	4.16	0.1672	0.1829	8.58	183.73	194.89	5.72	29.14	33.18	12.18
L6	0.2335	0.2252	-3.69	0.2084	0.2299	9.35	227.12	240.29	5.48	30.68	37.40	17.99
L7	0.2006	0.2095	4.25	0.1879	0.2264	17.01	191.17	198.98	3.92	28.01	34.46	18.72
L8	0.1795	0.2058	12.78	0.2632	0.2362	-11.43	200.50	343.88	41.70	30.94	35.71	13.37
L9	0.2260	0.2195	-2.96	0.1959	0.2160	9.31	210.77	205.93	-2.35	32.34	35.23	8.22
L10	0.2120	0.2232	5.02	0.1808	0.2133	15.24	205.05	204.64	-0.20	33.17	39.04	15.05
L11	0.2048	0.1904	-7.56	0.1740	0.1794	3.01	194.63	201.09	3.21	32.82	38.13	13.92
L12	0.1945	0.2174	10.53	0.1833	0.2329	21.30	198.49	208.91	4.99	31.23	36.90	15.36
L13	0.2200	0.2360	6.78	0.2429	0.2430	0.04	233.37	249.22	6.36	30.40	35.97	15.48
L14	0.2147	0.2078	-3.32	0.1763	0.1973	10.64	192.83	203.36	5.18	29.80	31.89	6.55
L15	0.1764	0.1997	11.67	0.1607	0.1858	13.51	180.74	197.98	8.71	30.12	33.69	10.62
L16	0.2443	0.2387	-2.35	0.1991	0.1993	0.10	189.56	197.03	3.79	33.58	36.29	7.45
L17	0.2069	0.2274	9.01	0.2053	0.2035	-0.88	207.70	227.69	8.78	32.07	35.49	9.63
L18	0.2233	0.2327	4.04	0.1856	0.2106	11.87	222.02	210.59	-5.43	34.01	37.89	10.23
L19	0.2093	0.2750	23.89	0.2305	0.2610	11.69	269.29	261.94	-2.81	31.81	34.19	6.96
L20	0.1887	0.2298	17.89	0.1694	0.1914	11.49	186.66	202.20	7.68	28.40	32.58	12.82
L21	0.1965	0.2601	24.45	0.2028	0.2494	18.68	214.12	200.07	-7.02	31.52	32.92	4.25
L22	0.2518	0.2682	6.11	0.2122	0.2227	4.71	217.79	212.54	-2.47	33.76	33.94	0.52
L23	0.2293	0.2461	6.83	0.2171	0.2057	-5.54	253.23	223.49	-13.31	34.39	38.54	10.77
L24	0.2171	0.2213	1.90	0.2228	0.2390	6.78	241.79	214.71	-12.62	34.23	37.60	8.97
L25	0.1860	0.2031	8.42	0.1628	0.1885	13.63	188.05	195.93	4.03	29.50	32.24	8.50
L26	0.1827	0.1964	6.98	0.1650	0.1943	15.08	185.24	193.71	4.37	32.55	33.46	2.70
2B587PW	0.2526	0.2515	-0.44	0.3216	0.2643	-21.68	299.49	285.97	-4.72	34.11	36.63	6.88
Mean	0.2095	0.2245	6.44	0.1988	0.2140	7.26	215.15	220.48	1.82	31.06	35.24	11.75

^1/^IEI = $\left(1-\frac{-Azo}{+Azo}\right)\;x\;100$

**Fig 1:**
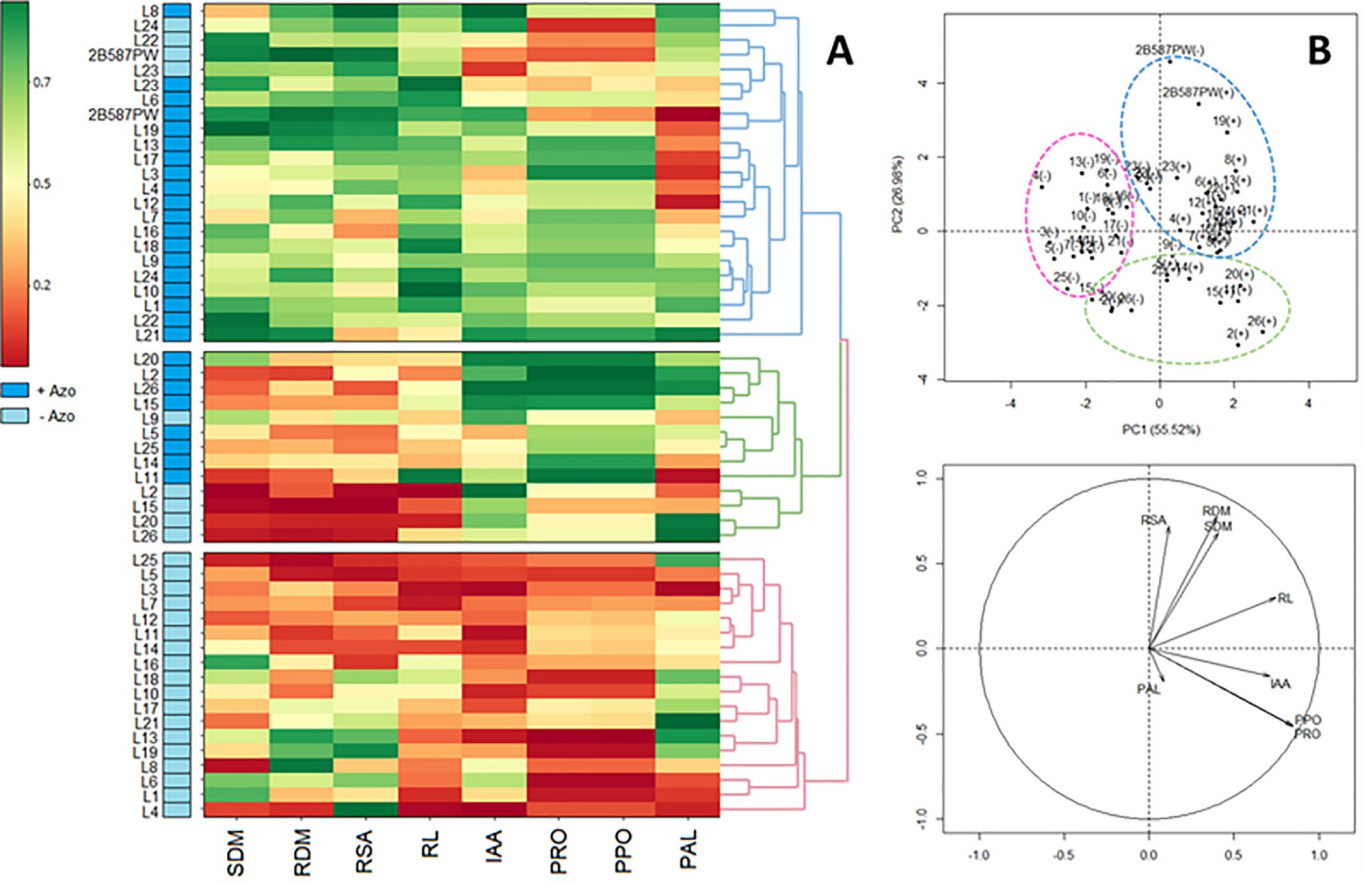
Heatmap (a) and principal component analysis (b) of the relationship between indole-3-acetic acid (IAA), total soluble protein (PRO), phenylalanine ammonia-lyase (PAL), polyphenoloxidase (PPO), total root length (RL), shoot dry mass (SDM), total root surface area (RSA) and root dry mass (RDM) evaluated in 27 maize genotypes inoculated (+Azo) and uninoculated (−Azo) with *Azospirillum brasilense* Ab-V5.

The formation of three large groups was detected by heatmap analysis (Fig 1A). Principal component analysis (PCA) explained 82.5% of the total variation by the first two components, and the resulting groups coincided with those of the heatmap (Fig 1B). Group I (blue) comprised most of the +Azo treatments, aside from the genotypes L22, L23, L24 and 2B587PW in condition −Azo. Thirteen inbred lines were clustered in group II (green), eight of which in condition +Azo and five in −Azo. On the other hand, group III (pink) consisted of 18 inbred lines in condition −Azo. In general, the mean genotype values of group I were highest for SDM, RDM, RSA and RL, and those of group II for IAA, PAL and PPO. On the other hand, the means of group III were the lowest for all evaluated traits.

### Experiment in Leonard pots

The predicted genotypic values under HN, LN and LN+Azo, as well as their respective IEI, are shown in Table 3. The highest general means were observed under HN for all evaluated traits except NUE, for which the overall mean was highest in condition LN+Azo. Positive mean values were observed for SDM (6.90%), RDM (5.45%) and NUE (27.99%), and negative values for RV (-3.98%). The IEI was positive for NUE for all evaluated genotypes, ranging from 3.99 (L17) to 49.31% (L12), except for the genotypes L16 (-14.23%) and 2B58PW (-22.97%). In general, the inbred lines L7, L8, L11, L18 and L23 stood out with highest IEI, with positive values for most evaluated traits.

**Table 3:** Predicted genotypic means of shoot dry mass (SDM), root dry mass (RDM), total root volume (RV), nitrogen use efficiency (NUE) and their respective inoculation efficiency indices (IEI) were evaluated in 27 maize genotypes grown in Leonard pots under high nitrogen (HN), low nitrogen (LN) and low nitrogen plus inoculation with *Azospirillum brasilense* Ab-V5 (LN+Azo).

Genotypes	SDM (g plant^-1^)				RDM (g plant^-1^)				RV (cm^3^ plant^-1^)				NUE (mg mg^-1^)			
	HN	LN+Azo	LN	IEI (%)^1^^/^	HN	LN+Azo	LN	IEI (%)	HN	LN+Azo	LN	IEI (%)	HN	LN+Azo	LN	IEI (%)
L1	0.36	0.20	0.20	-2.80	0.31	0.24	0.28	-15.76	3.66	3.63	4.57	-20.59	1.42	43.36	31.86	26.52
L2	0.25	0.18	0.17	3.28	0.20	0.20	0.19	7.19	3.30	3.03	3.24	-6.38	0.69	33.61	19.56	41.80
L3	0.26	0.18	0.18	3.90	0.23	0.25	0.21	20.32	3.45	3.54	3.62	-2.37	0.87	37.59	28.03	25.43
L4	0.40	0.24	0.20	20.72	0.22	0.22	0.20	9.43	3.96	3.42	3.67	-6.90	1.22	46.09	25.84	43.94
L5	0.32	0.19	0.19	3.75	0.25	0.26	0.25	1.70	5.06	3.86	3.90	-0.98	1.49	40.35	34.80	13.75
L6	0.27	0.22	0.20	8.24	0.28	0.23	0.22	3.97	3.77	3.49	4.44	-21.21	1.10	53.15	46.37	12.76
L7	0.30	0.25	0.20	25.95	0.23	0.22	0.22	0.32	4.04	3.31	3.57	-7.19	1.09	51.33	26.37	48.63
L8	0.31	0.26	0.24	8.68	0.24	0.23	0.21	9.11	3.61	3.93	3.78	4.03	0.89	58.90	35.68	39.42
L9	0.38	0.20	0.18	7.17	0.34	0.30	0.22	32.58	6.28	3.74	4.74	-21.10	1.92	44.07	22.94	47.95
L10	0.29	0.22	0.22	3.48	0.25	0.21	0.22	-4.15	3.51	3.69	4.14	-10.85	0.85	44.39	29.59	33.34
L11	0.27	0.23	0.19	19.59	0.26	0.22	0.20	13.45	3.38	3.46	3.43	0.74	0.85	42.13	22.36	46.93
L12	0.37	0.21	0.19	10.84	0.21	0.22	0.25	-9.14	5.34	3.80	4.01	-5.22	1.11	57.05	28.92	49.31
L13	0.42	0.23	0.21	10.56	0.24	0.27	0.23	18.85	4.87	4.01	4.07	-1.46	1.50	51.49	34.94	32.14
L14	0.33	0.19	0.18	3.09	0.23	0.21	0.23	-9.49	4.12	3.15	3.48	-9.32	1.46	37.66	25.17	33.17
L15	0.25	0.18	0.17	7.81	0.20	0.19	0.19	2.75	3.23	3.09	3.10	-0.39	0.68	30.42	19.11	37.18
L16	0.29	0.19	0.19	1.35	0.24	0.24	0.21	11.61	4.41	4.21	3.73	12.97	0.90	34.30	39.18	-14.23
L17	0.33	0.19	0.19	-3.88	0.26	0.21	0.24	-9.18	3.90	3.28	3.52	-7.00	1.39	33.80	32.45	3.99
L18	0.36	0.21	0.22	-4.67	0.28	0.32	0.27	19.56	4.20	4.36	3.84	13.41	1.19	54.82	42.44	22.58
L19	0.35	0.25	0.21	22.47	0.22	0.24	0.26	-6.75	4.30	4.10	4.22	-2.87	1.31	48.89	28.80	41.09
L20	0.34	0.22	0.23	-4.45	0.21	0.20	0.20	0.00	3.56	3.35	3.34	0.14	1.09	41.66	28.09	32.57
L21	0.50	0.20	0.18	14.05	0.26	0.25	0.19	28.07	4.68	3.20	3.18	0.66	1.71	34.34	22.46	34.60
L22	0.34	0.20	0.17	16.06	0.27	0.26	0.31	-14.82	5.77	4.81	5.04	-4.66	1.96	46.19	29.94	35.18
L23	0.30	0.21	0.19	10.22	0.29	0.28	0.21	37.15	3.84	4.52	4.32	4.45	1.15	49.43	27.74	43.88
L24	0.31	0.19	0.19	0.00	0.23	0.23	0.24	-4.60	4.54	3.58	3.96	-9.62	1.01	33.29	30.77	7.57
L25	0.26	0.20	0.20	-0.55	0.22	0.21	0.21	-1.01	3.16	3.39	3.39	-0.19	0.74	33.10	28.49	13.93
L26	0.28	0.18	0.18	0.28	0.21	0.21	0.20	4.75	3.72	3.24	3.30	-1.68	0.98	28.99	21.66	25.28
2B587PW	0.46	0.27	0.27	1.18	0.42	0.39	0.38	1.35	7.64	5.51	5.72	-3.76	3.53	67.62	83.15	-22.97
Mean	0.33	0.21	0.20	6.90	0.25	0.24	0.23	5.45	4.27	3.73	3.90	-3.98	1.26	43.63	31.36	27.99

^1/^IEI = $\left(1-\frac{LN}{LN+Azo}\right)\;x\;100$

**Fig 2:**
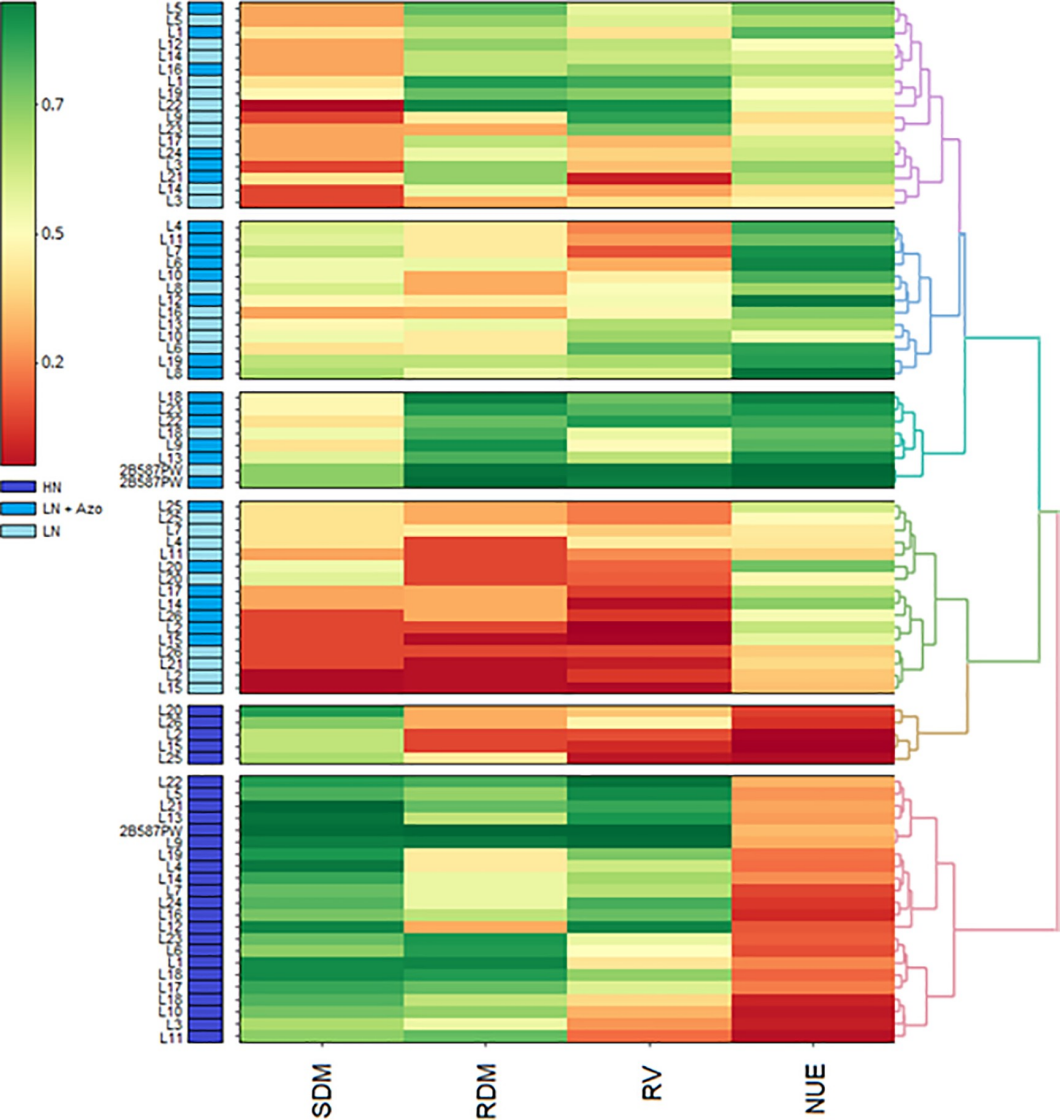
Heatmap of the relationship between total root volume (RV), root dry mass (RDM), shoot dry mass (SDM) and nitrogen use efficiency (NUE) evaluated in 27 maize genotypes under high nitrogen (HN), low nitrogen (LN) and low nitrogen plus inoculation with *Azospirillum brasilense* Ab-V5 (LN+Azo).

The heatmap was used to distinguish the lines in six groups (Fig 2). The genotypes under LN were distributed in the groups I (purple), II (dark blue), III (light blue) and IV (green), while genotypes under HN were allocated to groups V (yellow) and VI (pink). The inbred lines clustered in groups II and III had the highest mean values for NUE, especially those allocated in group II, in which the means were also high for the traits RDM and RV. Groups V and VI had the lowest NUE means; however, the inbred lines in group VI had high means for the other evaluated traits.

### Experiment in pots with substrate

The predicted genotypic values under HN, LN and LN+Azo, as well as their respective IEI, are shown in Table 4. The overall means were highest in the condition HN for the traits SDM and RDM, while in LN+Azo, the overall means were highest for RV and NUE. In relation to the IEI, positive general means were observed for all evaluated traits, ranging from 12.05 (NUE) to 26.03% (RV). In general, most of the genotypes had positive IEI values for the traits SDM, RDM, RV and NUE, mainly inbred lines L1, L6, L7, L8, L13 and L24.

**Table 4:** Predicted genotypic means of the traits shoot dry mass (SDM), root dry mass (RDM), total root volume (RV), nitrogen use efficiency (NUE) and their respective inoculation efficiency indices (IEI), evaluated in 27 maize genotypes grown in pots under high nitrogen (HN), low nitrogen (LN) and low nitrogen plus inoculation with *Azospirillum brasilense* Ab-V5 (LN+Azo).

Genotypes	SDM (g plant^-1^)				RDM (g plant^-1^)				RV (cm^3^ plant^-1^)				NUE (mg mg^-1^)			
	HN	LN+Azo	LN	IEI (%)^1^^/^	HN	LN+Azo	LN	IEI (%)	HN	LN+Azo	LN	IEI (%)	HN	LN+Azo	LN	IEI (%)
L1	4.02	3.07	2.54	21.14	2.03	1.86	1.50	23.89	20.94	16.67	15.89	4.91	9.97	76.10	62.97	17.25
L2	2.82	2.19	2.08	5.33	1.03	1.05	0.96	9.22	12.37	12.74	10.47	21.64	6.99	54.29	51.56	5.03
L3	3.41	2.56	2.23	14.84	1.13	1.23	1.02	21.35	13.33	12.43	10.20	21.85	8.45	63.46	55.28	12.89
L4	4.23	2.63	2.84	-7.46	1.36	1.10	1.17	-5.75	13.65	12.58	11.03	14.11	10.49	65.20	70.40	-7.98
L5	3.08	2.32	2.63	-12.01	1.10	1.21	1.08	11.45	13.18	13.70	10.89	25.83	7.64	57.51	65.20	-13.37
L6	3.26	2.78	2.18	27.69	1.30	1.53	1.12	35.62	13.83	15.04	11.69	28.66	8.08	68.92	54.04	21.59
L7	4.10	3.29	2.69	22.21	1.49	1.67	1.32	26.64	15.20	13.98	11.34	23.22	10.16	81.56	66.68	18.24
L8	3.94	2.94	2.44	20.68	1.21	1.56	1.05	48.59	14.43	16.25	11.52	41.07	9.77	72.88	60.49	17.00
L9	3.36	2.43	2.12	14.84	1.40	1.49	1.26	18.15	13.49	13.57	13.29	2.08	8.33	60.24	52.55	12.77
L10	3.17	2.48	2.02	22.67	1.33	1.31	0.93	40.81	14.23	13.45	10.34	30.09	7.86	61.48	50.08	18.54
L11	2.86	2.27	1.98	14.57	1.11	1.15	0.89	29.41	12.66	13.05	9.93	31.43	7.09	56.27	49.08	12.78
L12	3.60	2.38	2.19	8.56	1.58	1.34	1.00	34.01	12.52	15.42	12.66	21.73	8.92	59.00	54.29	7.98
L13	4.47	3.74	2.78	34.90	1.63	1.95	1.64	18.79	16.43	15.23	12.96	17.49	11.08	92.71	68.92	25.66
L14	2.90	2.34	2.14	9.23	1.15	1.73	0.98	76.54	17.00	14.45	9.67	49.45	7.19	58.01	53.05	8.55
L15	2.95	2.25	2.30	-2.26	1.06	1.08	0.95	13.76	11.74	14.65	9.53	53.76	7.31	55.78	57.02	-2.22
L16	3.03	2.29	2.36	-2.85	1.24	1.43	1.29	10.92	14.02	14.28	13.78	3.63	7.51	56.77	58.50	-3.05
L17	3.12	2.41	2.25	6.81	1.17	1.40	1.19	17.41	15.91	14.85	11.89	24.88	7.73	59.74	55.78	6.63
L18	3.31	2.60	2.33	11.31	1.27	1.63	1.22	33.82	14.68	15.90	12.08	31.65	8.21	64.45	57.76	10.38
L19	3.66	2.51	2.16	16.17	1.45	1.37	1.41	-3.18	13.05	12.91	11.19	15.33	9.07	62.22	53.55	13.93
L20	2.99	2.67	2.58	3.66	1.04	1.18	1.06	10.81	12.16	13.21	10.07	31.21	7.41	66.19	63.96	3.37
L21	3.21	2.85	2.28	24.93	1.19	1.28	1.03	24.43	12.80	13.34	10.62	25.66	7.96	70.65	56.52	20.00
L22	3.53	2.53	2.05	23.33	1.53	1.59	1.36	16.59	17.88	17.93	14.52	23.44	8.75	62.72	50.82	18.97
L23	3.47	2.36	2.10	12.51	1.69	1.26	1.10	14.04	15.53	13.84	12.44	11.26	8.60	58.50	52.06	11.01
L24	3.84	2.72	2.40	13.47	1.92	1.81	1.24	45.78	14.93	15.66	12.27	27.63	9.52	67.43	59.50	11.76
L25	3.75	2.46	2.21	11.03	1.78	1.46	1.15	27.07	12.93	14.13	10.76	31.38	9.30	60.98	54.79	10.15
L26	2.78	2.23	1.95	14.32	1.08	1.13	0.91	23.75	11.95	17.23	9.80	75.75	6.89	55.28	48.34	12.55
2B587PW	4.39	3.55	2.48	43.13	2.12	2.18	1.76	23.65	19.52	19.60	17.26	13.55	10.88	88.00	61.48	30.14
Mean	3.45	2.62	2.31	13.81	1.38	1.44	1.17	23.98	14.46	14.67	11.78	26.03	8.56	65.05	57.21	12.05

^1/^IEI = $\left(1-\frac{LN}{LN+Azo}\right)\;x\;100$

**Fig 3:**
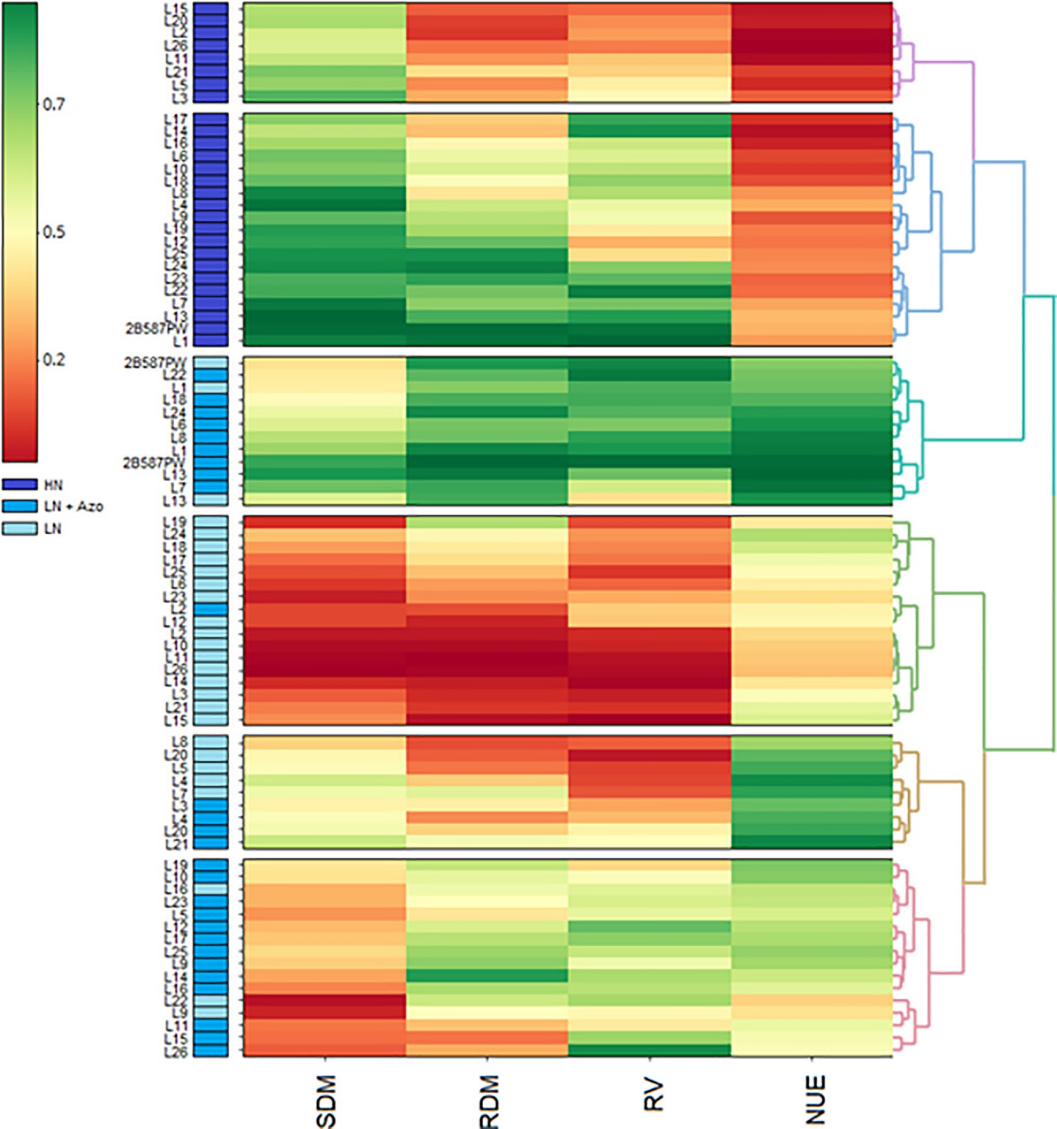
Heatmap of the relationship between total root volume (RV), root dry mass (RDM), shoot dry mass (SDM) and nitrogen use efficiency (NUE) evaluated in 27 maize genotypes under high nitrogen (HN), low nitrogen (LN) and low nitrogen plus inoculation with *Azospirillum brasilense* Ab-V5 (LN+Azo).

The heatmap showed the formation of six groups (Fig 3). The genotypes under HN were all allocated in groups I (purple) and II (dark blue). Group III (light blue) was formed by the genotypes in condition LN+Azo, except for the genotypes L1, L3 and 2B587PW at LN. With the exception of inbred line L2, group IV (green) consisted only of lines under LN, whereas the groups V (yellow) and VI (pink) were formed by inbred lines in the conditions LN and LN+Azo. In general, the genotypes under HN (groups I and II) had a lower NUE and higher SDM. Group III was characterized by the highest means for RDM, RV and NUE, while in group IV, the mean values for SDM, RDM and RV were the lowest. Group IV can be characterized by high means for NUE, and group VI by median values for all evaluated traits.

### Correlation between experiments

**Fig 4:**
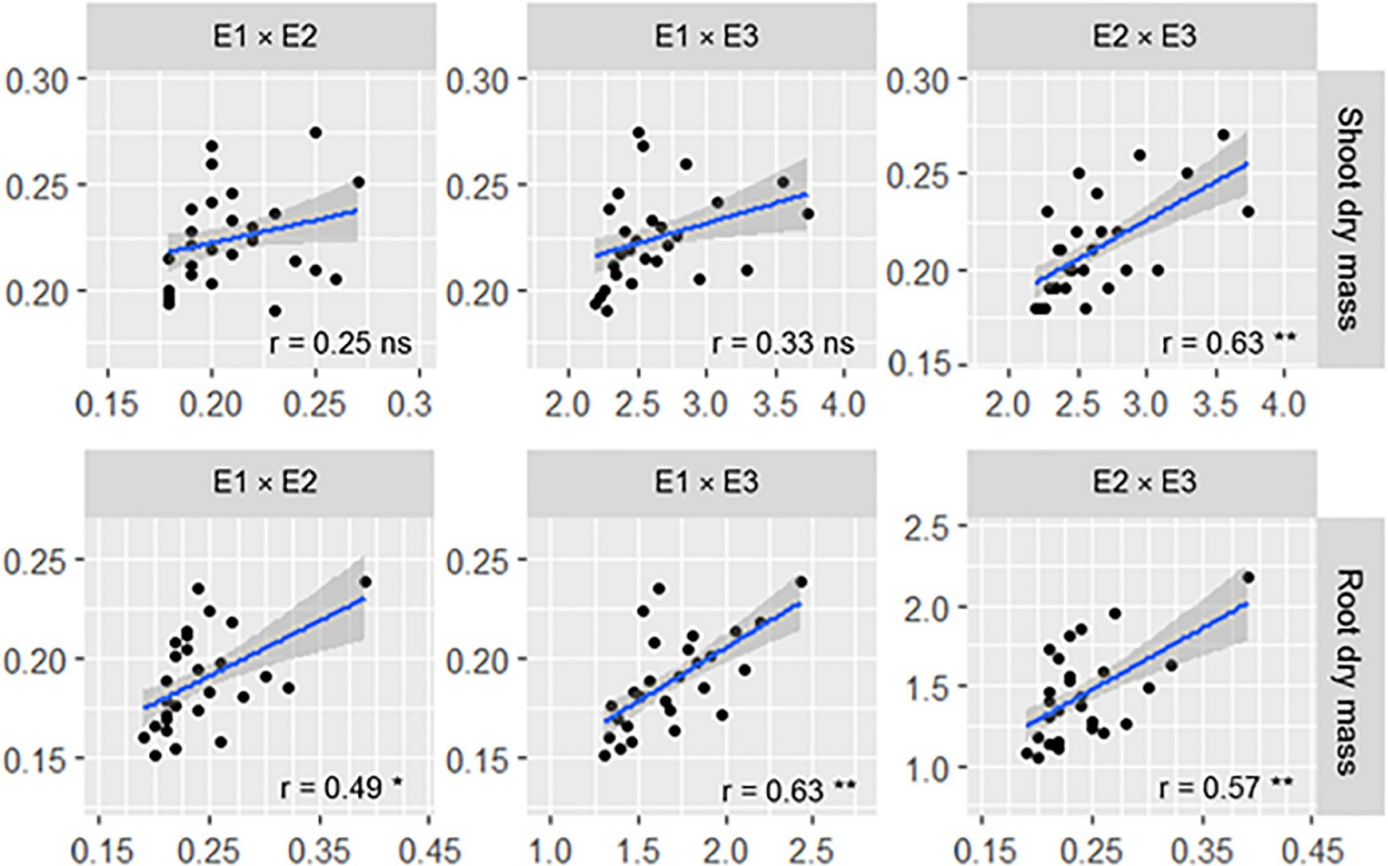
Pearson's correlation coefficients between the traits shoot and root dry mass between the experiments with maize on germination paper (E1), in Leonard pots (E2) and pots with substrate (E3). ns, * and ** = non-significant, significant at 5 and 1% probability by the t test, respectively.

By means of a correlation analysis between the experiments (Fig 4), a positive and significant correlation was observed between experiments E1 × E2 for trait RDM (r = 0.49*). Between the experiments E1 × E3, positive and significant correlations were found for RDM (r = 0.63**), as well as for E2 × E3 for SDM (r = 0.62**) and RDM (r = 0.57**).

## Discussion

The results of this study indicated that maize inoculation with *A*. *brasilense* Ab-V5 improved plant growth and biochemical traits and increased NUE under N limiting conditions. Metabolic changes in maize plants in response to *A*. *brasilense* inoculation were described previously, e.g., an improved root architecture [51], increase in plant biomass [52] and N assimilation [53], as well as mitigation of abiotic stresses [54–56]. In this way, the results show the powerful effect of *A*. *brasilense* inoculation on maize, mainly under limiting nutritional conditions, and also reinforce the importance of the plant microbiota as an extension of the maize genome to beat developmental restrictions under limiting-growth conditions [57].

In most maize genotypes inoculated with *A*. *brasilense* Ab-V5, the IAA concentration increased, possibly favoring plant growth and development. This beneficial effect can be related to the observed increases in the plant biomass and modifications on the root architecture in experiment E1. The initial effect of *Azospirillum* inoculation on the promotion of seedling growth can be mimicked a phytohormone treatment [58,59]. However, modifications in the plant development pattern during an extensive growth period require the uninterrupted entry of exogenous phytohormones, which occurs when *Azospirillum* colonizes the plants [21]. Although the IAA biosynthesis by *Azospirillum* is influenced by endogenous and exogenous factors, it is produced during all phases of bacterial development, which is a highly relevant characteristic for plant growth promotion, since benefits can already be observed in the first days or months after inoculation [60]. According to Bashan and de-Bashan [23], phytostimulation of *Azospirillum* by means of IAA biosynthesis is extremely important in the early growth stages (germination and initial seedling growth) and is considered complementary to other mechanisms at more advanced plant growth stages.

Increases in the traits related to plant growth were also observed in the experiments E1 and E2, reinforcing the role of *A*. *brasilense* in promoting structural changes that are essential for plant growth and development. Changes in the root system of *Azospirillum*-inoculated plants have already been observed, such as root elongation [53,61], development of lateral and adventitious roots [62,63] and root hair development [64,65]. These modifications were associated to increases in plant biomass and nutrient uptake, increasing the tolerance to limiting nutritional conditions [24,29]. The structural morphological changes in inoculated plants are partly caused in response to phytohormone production and release by *Azospirillum* [15]. The IAA, for example, is related to the division, extension and differentiation of plant cells and tissues and closely linked to the differentiation of the vascular system of plants [66,67]. In a study on the effect of *A*. *brasilense* Ab-V5 on maize, Calzavara et al. [52] observed a higher number of elements of the metaxylem of inoculated plants than of the control plants. This resulted in a thicker vascular cylinder of the plants, which is favorable for water and nutrient transport, resulting in higher root and shoot biomass production.

Although the efficiency of PGPB inoculation may vary according to the plant genotype, bacterial strain and environmental conditions [68], the influence of N fertilization management on inoculation efficiency has been considered more relevant [69]. According to Rozier et al. [70], in a study on the effect of the different levels of N fertilization associated with *A*. *lipoferum* inoculation, N fertilization induced higher maize grain yields. However, no influence of *A*. *lipoferum* inoculation on this increment was detected, suggesting that these technologies are not additive. In the same context, a meta-analysis of the effect of *Azospirillum* spp. on maize yield showed a mean increase of 651.58 kg ha^-1^ in inoculated over uninoculated treatments [71]. However, the same study observed a strong influence of the levels of N topdressing (absence, low, moderate and high) on inoculation efficiency, since the positive effects of inoculation were only significant in the absence of N topdressing, which confirms the theory of non-additivity of the two technologies. Thus, the use of *Azospirillum* as biofertilizer can be considered a promising technology, in particular under N stress [31,72,73].

Nitrogen limitation in maize can drastically reduce the photosynthetic activity of plants [74] and interfere with the transcription of genes related to the N and C metabolisms, causing a reduction in biomass production and, consequently, limiting grain yields [75]. In this sense, plants with a higher NUE can reduce the damages caused by N limitation, since they require a smaller amount of this nutrient for biomass and/or grain production [76]. In the experiments E2 and E3, a higher NUE of the inoculated genotypes could be observed in relation to the uninoculated genotypes, indicating that inoculation with *A*. *brasilense* Ab-V5 raised NUE under LN availability. In experiment E2, the genotypes with highest NUE were the same in the LN and LN+Azo conditions. However, this coincidence was not observed in experiment E3, indicating a differentiated NUE between genotypes under *A*. *brasilense* Ab-V5 inoculation.

Nitrogen use efficiency does not only depend on an efficient N uptake from the soil, but also on the internal transport, storage, recycling, remobilization and growth stage of the plants [77]. Several strategies have been used to improve NUE of plants [11,78]. However, since PGPB have the capacity to promote plant growth and nutrient uptake, they can be considered a promising solution to increase the efficiency of nutrient use, which is reinforced by the results obtained in this study. An increase in the efficiency of nutrient use by plants has been reported for several PGPB genera, since they are not only able to fix N_2_, but are also capable of solubilizing mineral and/or organic nutrients of the soil [14,79]. A meta-analysis addressing the benefits of PGPB in relation to NUE in several plant species identified a mean increment of 5.8±0.6 kg grain per kg fertilizer, reinforcing biofertilizers as a promising technology under limiting cultivation conditions [80].

In an evaluation of the response of greenhouse maize to *A*. *brasilense* inoculation in clayey and sandy soil, Ferreira et al. [81] stated a positive response of maize to inoculation. However, these responses were dependent on the soil type and substrate, since increases in the evaluated traits were only observed in clayey soil. Similarly, Mehnaz et al. [24] observed differentiated responses among maize varieties inoculated with *A*. *brasilense* or *A*. *lipoferum* in pots with sand or soil, allowing the conclusion that, aside from the maize genotype and *Azospirillum* species, the type of substrate may also influence the effect of inoculation. In this study, although the experimental conditions of evaluation were contrasting, the observed results were similar under the three experimental conditions (E1, E2 and E3), mainly for RDM.

In general, the inbred lines L7 and L8 were the most responsive in relation to the efficiency of *A*. *brasilense* Ab-V5 inoculation, whereas line L16 was least responsive to inoculation. The identification of contrasting genotypes regarding inoculation response is fundamental in studies on the plant–*Azospirillum* interaction. In a population of 114 double haploid wheat (*Triticum aestivum* L.) lines, derived from the cross between two parents contrasting in terms of root adhesion of *A*. *brasilense*, De León et al. [82] identified six quantitative trait loci (QTL) responsible for 23.1% of the phenotypic variation of this trait. Among these, a QTL of greater effect was found to be responsible for 8.63% of this variation. The identification of genes/QTLs related to the plant–*Azospirillum* interaction may provide numerous molecular markers which, in the future, may be used in marker-assisted selection (MAS) for a successful plant–*Azospirillum* interaction, contributing to the breeding of plants associated with PGPB.

## Supporting information

S1 Data Data from the experiment on germination paper. Indole-3-acetic acid (IAA); total soluble proteins (PRO); polyphenoloxidase (PPO); phenylalanine ammonia-lyase (PAL); total root length (RL); shoot dry mass (SDM); total root surface area (RSA); root dry mass (RDM); without (–Azo) and with inoculation of *Azospirillum brasilense* (+Azo).
(XLSX)

S2 Data Data from the experiment in Leonard pots. Total root volume (RV); root dry mass (RDM); shoot dry mass (SDM); nitrogen use efficiency (NUE); high nitrogen (HN); low nitrogen (LN) and low nitrogen plus inoculation with *Azospirillum brasilense* (LN+Azo).
(XLSX)

S3 Data Data from the experiment in pots with substrate. Total root volume (RV); root dry mass (RDM); shoot dry mass (SDM); nitrogen use efficiency (NUE); high nitrogen (HN); low nitrogen (LN) and low nitrogen plus inoculation with *Azospirillum brasilense* (LN+Azo).
(XLSX)
